# Impact of Allergic Contact Dermatitis on Health‐Related Quality of Life: A Cross‐Sectional Case–Control Study in a Spanish Population

**DOI:** 10.1111/cod.70116

**Published:** 2026-02-22

**Authors:** Francisco José Navarro‐Triviño, Álvaro Prados‐Carmona, Ricardo Ruiz‐Villaverde, María Isabel Peralta‐Ramírez

**Affiliations:** ^1^ University of Granada Granada Spain; ^2^ Department of Contact Eczema and Immunoallergic Diseases, Dermatology University Hospital San Cecilio Granada Spain; ^3^ Instituto Biosanitario de Granada (ibs.Granada) Granada Spain; ^4^ Department of Dermatology University Hospital San Cecilio Granada Spain; ^5^ Escuela Internacional de Posgrado, University of Granada Granada Spain; ^6^ Mind, Brain and Behavior Research Center (CIMCYC) University of Granada Granada Spain; ^7^ Department of Personality, Assessment and Psychological Treatment, Faculty of Psychology University of Granada Granada Spain

**Keywords:** allergic contact dermatitis, DLQI, EQ‐5D‐5L, health‐related quality of life, Skindex‐29

## Abstract

**Background:**

Allergic contact dermatitis (ACD) is a chronic inflammatory skin disorder associated with substantial impairment in quality of life (QoL). Few studies have comprehensively assessed the multidimensional impact of ACD using validated QoL instruments and healthy controls.

**Objectives:**

To evaluate the impact of ACD on QoL compared to a control group and to explore the association between clinical variables and patient‐reported outcomes.

**Methods:**

This cross‐sectional study included 225 patients with confirmed ACD (positive and clinically relevant patch tests) and 225 healthy controls. All participants completed the *Dermatology Life Quality Index* (DLQI), the EuroQoL‐5D (EQ‐5D‐5L) and the Skindex‐29. Disease severity was assessed using the modified *Investigator's Global Assessment* (mIGA). Statistical analyses included nonparametric Mann–Whitney U tests for between‐group comparisons, correlation analyses, and multivariate linear and ordinal regression models to identify predictors of quality‐of‐life impairment.

**Results:**

Patients with ACD showed significantly greater impairment across all QoL measures compared to controls (*p* < 0.001). Pruritus was the most frequently reported symptom (45.0%), and emotional distress and functional limitations were prominent. Higher mIGA scores were significantly associated with poorer QoL across all instruments.

**Conclusions:**

ACD has a marked negative impact on multiple dimensions of QoL, comparable to that observed in other chronic dermatoses. These findings underscore the importance of integrating standardised QoL assessments into the routine management of ACD and support the adoption of multidimensional approaches in both clinical evaluation and therapeutic decision‐making, while also highlighting the relevance of psychosocial screening as an essential component of comprehensive patient care.

## Summary

- What is already known?
  ○ Allergic contact dermatitis (ACD) is a frequent inflammatory skin disease with a relevant impact on patients' daily life.
  ○ Previous studies assessing quality of life (QoL) in ACD have mainly relied on single instruments, specific anatomical locations, or selected populations, often without healthy control groups.
- What does this study add?
  ○ This study provides a comprehensive, multidimensional evaluation of QoL in ACD using three validated instruments (DLQI, EQ‐5D‐5L and Skindex‐29) and a matched healthy control group.
  ○ Patients with ACD showed significantly impaired QoL across symptomatic, emotional, functional and global health domains compared with controls.
  ○ Disease severity was a major determinant of quality‐of‐life impairment, supporting the routine use of standardised patient‐reported outcomes in the clinical management of ACD.

## Graphical abstract

Allergic contact dermatitis markedly impairs quality of life (QoL) across symptoms, functioning and emotional domains. Using DLQI, Skindex‐29 and EQ‐5D‐5L, our study demonstrates a substantial multidimensional burden and highlights the need for routine, patient‐centred QoL assessment in clinical management.

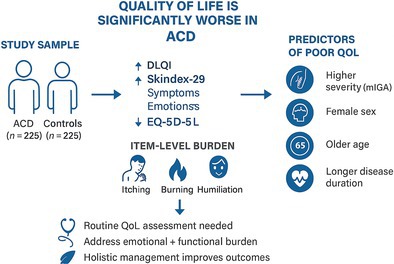

## 1 Introduction

Allergic contact dermatitis (ACD) is an inflammatory skin disorder that affects approximately 15%–20% of the general population [1] and accounts for 4%–7% of dermatology consultations [2]. This condition entails considerable public health and socioeconomic burdens [3], due to its variable course and significant impact on work performance, particularly in occupational cases with a high risk of chronicity [4].

In recent years, the increasing chronicity of dermatological conditions has highlighted the central role of quality of life (QoL) assessment in both clinical research and routine practice.

Validated instruments such as the Dermatology Life Quality Index (DLQI) [5], the EuroQoL 5D (EQ‐5D) [6] and the Skindex‐29 [7] are now widely recognised as standard tools in dermatology. They enable a comprehensive and patient‐centred understanding of disease burden. The impact of skin diseases on QoL has been shown to rival that of severe systemic conditions. Like other chronic inflammatory dermatoses, such as psoriasis [8], atopic dermatitis (AD) [9], or cutaneous lupus erythematosus [10], ACD also exerts a substantial negative effect on patients' QoL.

Beyond their diagnostic value, patch tests have also contributed to improving the QoL of patients with ACD by enabling targeted strategies for allergen avoidance [11]. Studies have shown that DLQI scores improve after patch testing [12], particularly in occupational cases and hand eczema [13]. In addition, the perception of the disease and emotional distress have been identified as important factors affecting QoL in ACD [14]. However, most of the available findings are still limited to occupational environments and localised forms of disease, such as hand eczema.

Despite the proven impact of ACD, existing studies have several limitations: many are restricted to specific anatomical areas, do not have healthy control groups, or rely on individual instruments to measure QoL. While some previous studies have used the DLQI to assess QoL in ACD, to our knowledge, none have employed a multidimensional evaluation using three validated instruments nor included a healthy control group for comparison.

Therefore, this study aimed to evaluate the impact of ACD on QoL compared with healthy controls and to examine how disease severity and duration relate to different QoL dimensions.

## 2 Materials and Methods

This cross‐sectional study included patients referred to the immunoallergy department of dermatology at a tertiary university hospital in Spain between January 2021 and December 2023. Healthy control subjects were primarily selected from among the patients' companions using a convenience sampling method. This cross‐sectional study was conducted and reported in accordance with the STROBE (Strengthening the Reporting of Observational Studies in Epidemiology) guidelines [15] and the recent STROBE‐Equity extension, which enhances reporting of health equity considerations in observational research [16]. The completed STROBE checklist is available as Supporting Information.

### 2.1 Patients

The study included 450 participants: 225 with ACD and 225 healthy controls. The sample size was determined based on previous studies assessing the psychosocial and quality‐of‐life impact of chronic dermatoses, assuming a significance level of 0.05 and a statistical power of 80% (Cohen's *d* = 0.5), ensuring sufficient power to detect medium effect sizes and reduce the risk of Type I and Type II errors.

### 2.2 Sample Size Calculation

Sample size was estimated a priori using G*Power 3.1 (Universität Düsseldorf, Germany). Based on previous studies evaluating the psychosocial and quality‐of‐life impact of chronic dermatoses, we assumed a medium effect size (Cohen's d = 0.5), a two‐tailed significance level (α = 0.05) and a statistical power (1 − β = 0.80). This calculation indicated a minimum requirement of 210 participants per group to detect medium differences in quality‐of‐life outcomes between patients with ACD and healthy controls.

To ensure adequate power for secondary and multivariate analyses (adjusting for age, sex, disease severity and duration), we slightly inflated the target sample, enrolling 225 patients with ACD and 225 healthy controls. The final sample size, therefore, exceeded the minimum required to achieve 80% power, minimising the risk of both Type I and Type II errors.

#### 2.2.1 Inclusion and Exclusion Criteria

Eligible participants were adults (≥ 18 years) who were able to read and write in Spanish.

Inclusion criteria and methodological procedures were based on previous studies that evaluated the psychosocial and quality‐of‐life impact of ACD and other chronic dermatoses using validated instruments [12, 17]. These studies applied similar inclusion criteria—adult patients with clinically confirmed ACD through standardised patch testing—and comparable instruments to assess health‐related QoL, ensuring methodological consistency with established literature.

Patients were included in the ACD group only if they had a confirmed diagnosis of exclusive ACD, established by positive patch test results to clinically relevant allergens. Individuals were excluded if they had intellectual disability, a diagnosed psychiatric disorder, current or past use of psychotropic medication, or the presence of any other dermatologic condition, such as irritant contact dermatitis, atopic dermatitis, psoriasis, or seborrheic dermatitis.

Controls were recruited among patients' relatives or companions attending the same dermatology clinic. This approach was chosen to enhance sociodemographic comparability between groups, minimise variability related to environmental or occupational exposure, and ensure the practical feasibility of recruitment within the same healthcare setting.

#### 2.2.2 Variables of Interest

Sociodemographic and clinical data were collected through structured clinical interviews. The diagnosis of ACD was confirmed by patch testing, following the standards established by the European Society of Contact Dermatitis (ESCD) [18].

### 2.3 Assessment Instruments

A semi‐structured interview was used to collect key sociodemographic and clinical variables. To assess QoL, three validated instruments adapted to the Spanish population were administered: the *Dermatology Life Quality Index* (DLQI), the *EuroQol 5‐Dimensions* (EQ‐5D‐5L) and the Skindex‐29.

These instruments were selected for their complementary profiles. The DLQI is widely used for clinical monitoring. The EQ‐5D‐5L provides a generic measure of health‐related QoL. The Skindex‐29 offers a multidimensional evaluation, capturing emotional and functional aspects specific to skin diseases. This combination allows for a comprehensive assessment of both general and dermatology‐specific QoL dimensions.

Dermatology Life Quality Index (DLQI) [19]: The DLQI is a widely used 10‐item questionnaire specifically designed to measure the impact of skin diseases on patients' daily lives. Each item is scored on a 4‐point Likert scale (0 = not at all to 3 = very much), with total scores ranging from 0 to 30; higher scores indicate greater impairment. The Spanish version has demonstrated good internal consistency (Cronbach's alpha ≈ 0.91) and validity in dermatological populations.

EuroQol 5‐Dimensions (EQ‐5D‐5L) [20]: The EQ‐5D‐5L is a generic health‐related QoL instrument that assesses five dimensions: mobility, self‐care, usual activities, pain/discomfort and anxiety/depression. Each dimension includes five response levels (ranging from “no problems” to “extreme problems”) and a visual analogue scale (VAS) from 0 to 100 to rate overall perceived health. The validated Spanish version has demonstrated adequate psychometric properties, with a Cronbach's alpha of 0.90 in the general population.

Skindex‐29 [7]: The Skindex‐29 is a dermatology‐specific questionnaire composed of 29 items divided into three domains: Symptoms, Emotions, and Functioning. Responses are rated on a 5‐point scale, and scores are linearly transformed to a 0–100 scale for each domain, with higher scores indicating greater impairment. The Spanish adaptation of the Skindex‐29 has shown high internal consistency (Cronbach's alpha > 0.85) and robust psychometric properties.

#### 2.3.1 Severity Assessment

The mIGA is a clinician‐rated scale used to assess the global severity of ACD at the time of clinical evaluation. It is a 5‐point ordinal scale ranging from 0 to 4, where 0 = clear, 1 = almost clear, 2 = mild, 3 = moderate and 4 = severe disease. The assessment considers key clinical signs such as erythema, infiltration, vesiculation, scaling and the overall extent of skin involvement. This tool has been previously validated for clinical research in ACD, including its use by Korkmaz et al. [21] to assess disease progression and treatment outcomes.

#### 2.3.2 Procedure

The eligible individuals were identified during their initial consultation at the immunoallergy department of a specialist dermatology clinic. After confirming that they met the inclusion criteria, patients were informed about the study's objectives and provided written informed consent prior to data collection.

During the initial examination, in which patients with clinically active lesions were examined, participants in the ACD group completed questionnaires on QoL (DLQI, Skindex‐29 and EQ‐5D‐5L). During the same examination, the severity of the disease was assessed by a dermatologist using the modified Investigator's Global Assessment (mIGA) scale. A patch test was then performed as part of the diagnostic evaluation. Only patients who received positive and clinically relevant patch test results were included in the final analysis of the ACD group. Demographic and clinical data were collected during this visit through structured interviews. Information on disease duration was obtained from the patient's electronic medical record.

Controls provided informed consent and completed the same questionnaires in a single session.

The assessment for both groups was conducted in a private and standardised environment and each session lasted approximately 30 min.

#### 2.3.3 Ethical Considerations

All participants provided written informed consent before inclusion in the study, following the Declaration of Helsinki (World Medical Association, 2013) and the European Union Directive on Good Clinical Practice (Directive 2005/28/EC). The study protocol was approved by the Institutional Research Ethics Committee (DERM_HUSC_‐2021).

### 2.4 Statistical Analysis

Descriptive statistics were used to summarise sociodemographic and clinical variables, expressed as means and standard deviations for continuous variables, and as frequencies and percentages for categorical variables. Before selecting statistical tests, all variables were examined to ensure they met the distributional assumptions. Between‐group comparisons (ACD vs. controls) for ordinal questionnaire data (DLQI and Skindex‐29) were performed using the Mann–Whitney U test, whereas categorical variables were compared using chi‐square tests. Associations between sociodemographic or clinical variables and QoL measures were explored using Spearman or Pearson correlation coefficients, as appropriate. To identify predictors of QoL impairment, multivariate analyses were conducted using linear regression models for continuous outcomes and ordinal regression models (proportional odds model) for ordinal outcomes. These models were adjusted for age, sex, disease duration and disease severity (mIGA). Missing data were handled using complete‐case analysis. Given the exploratory design of the study, no correction for multiple comparisons (e.g., Bonferroni or FDR) was applied; therefore, results should be interpreted with caution in light of potential Type I error inflation. A *p*‐value < 0.05 was considered statistically significant. All analyses were conducted using IBM SPSS Statistics version 27.0 (IBM Corp., Armonk, NY, USA).

## 3 Results

### 3.1 Sample Description

A total of 450 participants were enrolled in the study, comprising 225 patients with ACD and 225 healthy individuals serving as the control group. There were no statistically significant differences between groups in sociodemographic variables (all *p* > 0.05) (Table 1).

**TABLE 1:** Sociodemographic and clinical characteristics of participants.

Variable	ACD group (*n* = 225)	Control group (*n* = 225)	*p*
Age, mean (SD)	33.01 (4.56)	33.56 (4.33)	0.468
Sex, *n* (%)			0.611
Women	152 (67.6)	157 (69.8)	
Men	73 (32.4)	68 (30.2)	
Marital status, *n* (%)			0.953
Single	76 (33.8)	80 (35.6)	
Married	117 (52.0)	111 (49.3)	
Divorced	23 (10.2)	24 (10.7)	
Widowed	9 (4.0)	10 (4.4)	
Educational level, *n* (%)			0.592
Basic education	16 (7.1)	18 (7.8)	
Primary education	19 (8.4)	19 (8.4)	
Secondary education	38 (16.9)	32 (14.2)	
High school diploma	39 (17.3)	41 (18.2)	
University studies	113 (50.2)	115 (51.1)	
Employment status, *n* (%)			0.214
Employed	138 (61.3)	124 (55.1)	
Clinical variables (ACD group only)			
Disease duration, months, mean (SD)	68.26 (110.0)	—	—
Emergency visits (last 12 months), mean (SD)	1.61 (2.81)	—	—
≥ 1 emergency visit, *n* (%)	92 (40.9)	—	—
Disease severity (mIGA), mean (SD)	2.31 (1.06)	—	—
Functional limitation (NRS), mean (SD)	6.29 (2.90)	—	—
Concern about disease (NRS), mean (SD)	7.18 (2.70)	—	—
Self‐perceived severity (NRS), mean (SD)	5.37 (3.02)	—	—

Abbreviations: ACD, allergic contact dermatitis; mIGA, modified Investigator's Global Assessment; NRS, numeric rating scale; SD, standard deviation.

In the ACD group, mean disease duration was 68.26 months (SD = 110.0). In the previous 12 months, 40.89% of patients reported at least one ACD‐related emergency department visit (mean = 1.61, SD = 2.81). Mean disease severity according to the mIGA was 2.31 (SD = 1.06), corresponding to mild‐to‐moderate disease.

Patients reported substantial burden in daily functioning and emotional distress. The mean NRS score for functional limitation was 6.29 (SD = 2.90), while concern about the disease scored 7.18 (SD = 2.70). Additionally, self‐perceived disease severity showed an average score of 5.37 (SD = 3.02), reflecting a moderate subjective burden of disease. Group comparisons for NRS items were conducted using Mann–Whitney U tests.

### 3.2 Quality of Life Differences Between ACD Patients and Healthy Controls

#### 3.2.1 Dermatology Life Quality Index (DLQI)

**FIGURE 1:**
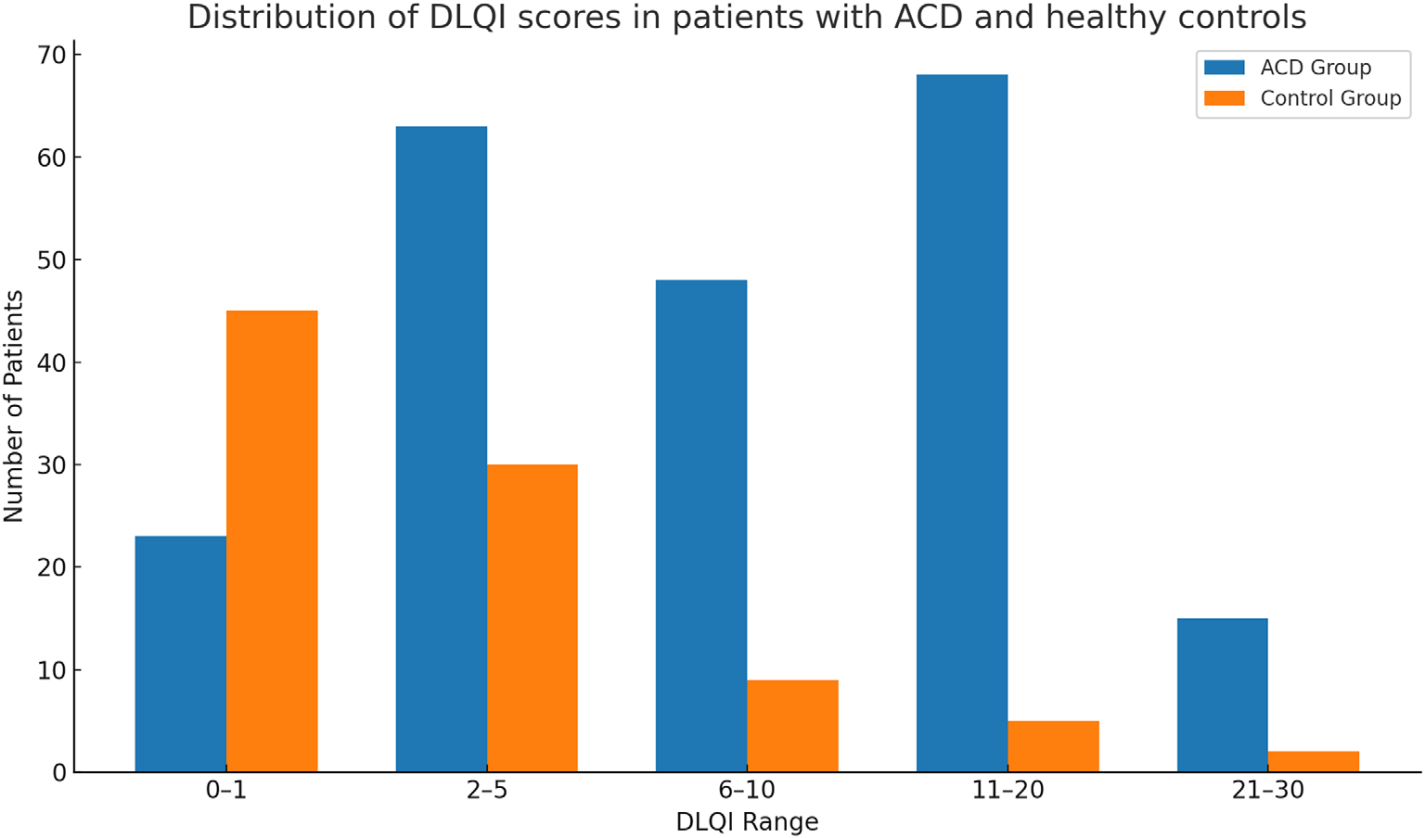
Distribution of Dermatology Life Quality Index (DLQI) score ranges in patients with Allergic Contact Dermatitis (ACD) and healthy controls. Score ranges correspond to: 0–1 (no effect), 2–5 (small effect), 6–10 (moderate effect), 11–20 (very large effect) and 21–30 (extremely large effect).

Patients with ACD had significantly higher DLQI scores (Mean = 8.72, SD = 6.80) than controls (Mean = 1.39, SD = 3.57) (*p* < 0.001). Notably, 57.78% of patients in the ACD group experienced a moderate‐to‐severe impact on QoL. The distribution of DLQI scores across severity categories is shown in Figure 1, highlighting the increased burden in the ACD group. An item‐level analysis of the DLQI revealed significantly higher scores for all individual items among ACD patients compared with controls (Table S1; Figure S1). The greatest differences were observed for items related to symptoms (“itchy, sore, painful, or stinging skin”) and emotional impact (“feeling embarrassed or self‐conscious”), followed by daily activities such as shopping and housework. These findings highlight the multidimensional burden of ACD, affecting both physical symptoms and psychosocial domains.

#### 3.2.2 EuroQol 5‐Dimensions (EQ‐5D‐5L)

**FIGURE 2:**
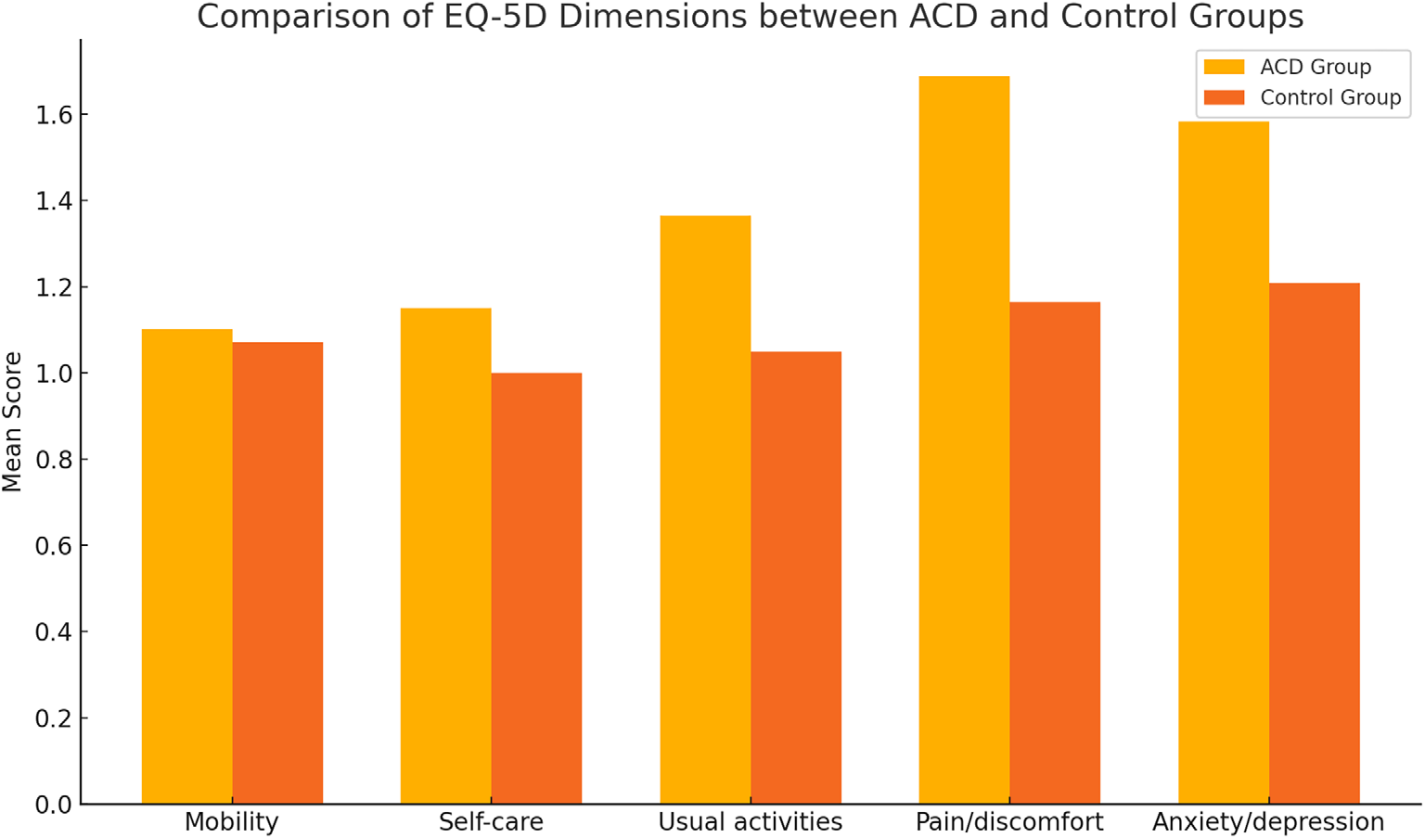
Comparison of mean scores across the five EQ‐5D‐5L dimensions in patients with allergic contact dermatitis (ACD) and healthy controls. Each dimension is rated on a 5‐point ordinal scale, where 1 indicates no problems, 2 slight problems, 3 moderate problems, 4 severe problems and 5 extreme problems or complete inability to perform the activity.

**FIGURE 3:**
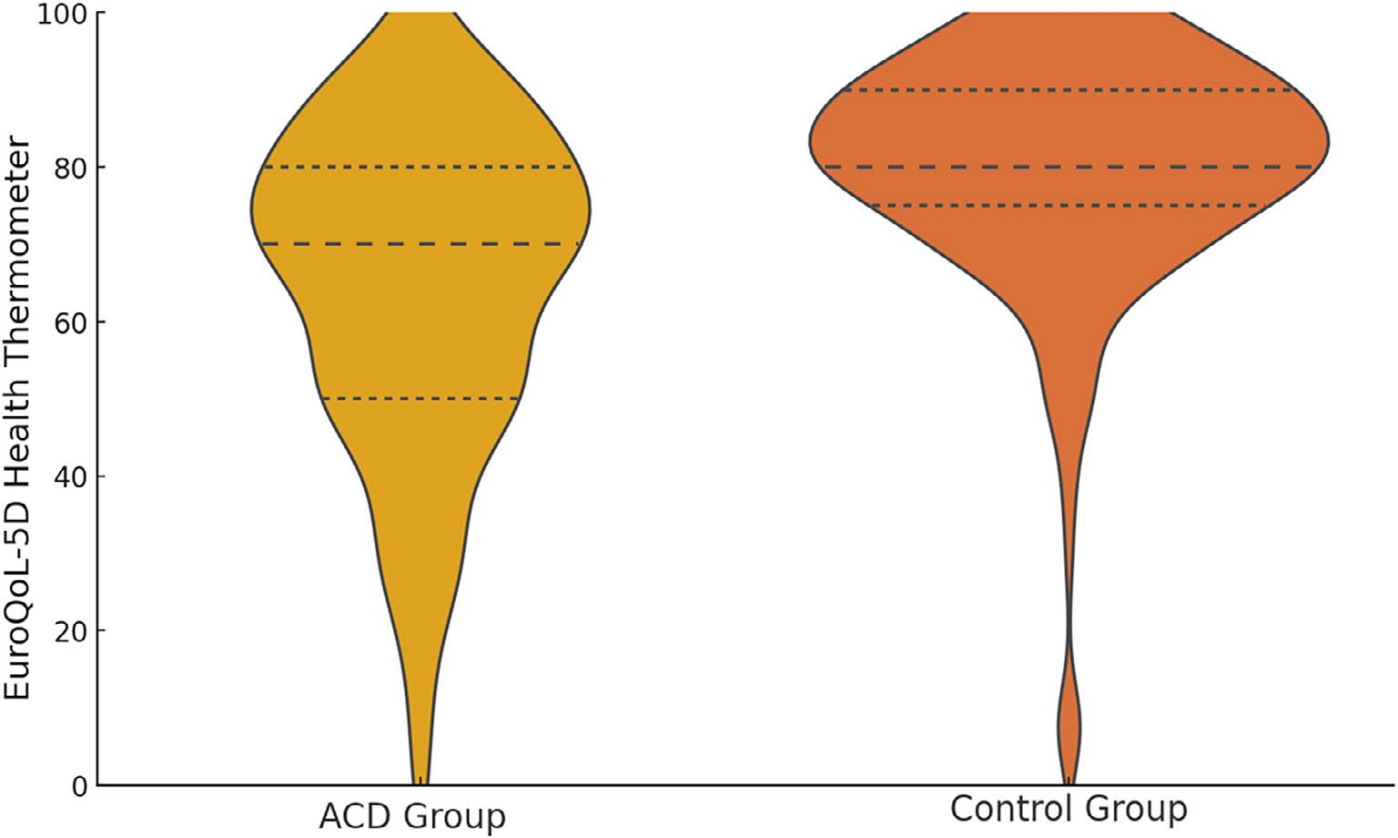
Distribution of health thermometer scores (EuroQoL‐5D VAS) in the ACD and control groups.

Table 2 presents the comparative analysis of EuroQoL‐5D dimensions between patients with ACD and the control group. EQ‐5D index scores were significantly lower in ACD patients (mean = 0.867, SD = 0.111) than in healthy controls (mean = 0.962, SD = 0.060; *p* < 0.001), indicating a reduced health‐related QoL. This difference is further reflected in Figure 2, which shows the proportion of participants reporting problems across each of the five EQ‐5D dimensions. Additionally, lower scores on the EQ VAS among ACD patients illustrate poorer self‐perceived health (Figure 3).

**TABLE 2:** Comparative analysis of EuroQoL‐5D dimensions between patients with ACD and the control group.

Variable	ACD group (mean ± SD)	Control group (mean ± SD)	Mean difference (95% CI)	*t*	*p*
Mobility	1.10 ± 0.304	1.07 ± 0.258	0.031 (−0.021 to 0.083)	1.172	0.242
Self‐care	1.15 ± 0.371	1.00 ± 0.000	0.151 (0.102 to 0.200)	6.107	< 0.001
Usual activities	1.36 ± 0.518	1.05 ± 0.216	0.316 (0.242 to 0.389)	8.433	< 0.001
Pain/discomfort	1.69 ± 0.620	1.16 ± 0.372	0.524 (0.430 to 0.619)	10.878	< 0.001
Anxiety/depression	1.58 ± 0.608	1.21 ± 0.429	0.373 (0.276 to 0.471)	7.529	< 0.001
EQ‐5D index value	0.867 ± 0.111	0.962 ± 0.060	−0.095 (−0.112 to −0.079)	−11.297	< 0.001
General health perception (past 12 months)	0.95 ± 0.739	0.69 ± 0.525	0.258 (0.139 to 0.377)	4.263	< 0.001
Health status thermometer (0–100)	64.79 ± 21.05	79.85 ± 15.86	−15.06 (−18.51 to −11.60)	−8.569	< 0.001

Abbreviations: ACD, allergic contact dermatitis; CI, confidence interval; EQ‐5D‐5L, EuroQol 5‐Dimensions 5‐Levels; SD, standard deviation.

#### 3.2.3 Skindex‐29

**FIGURE 4:**
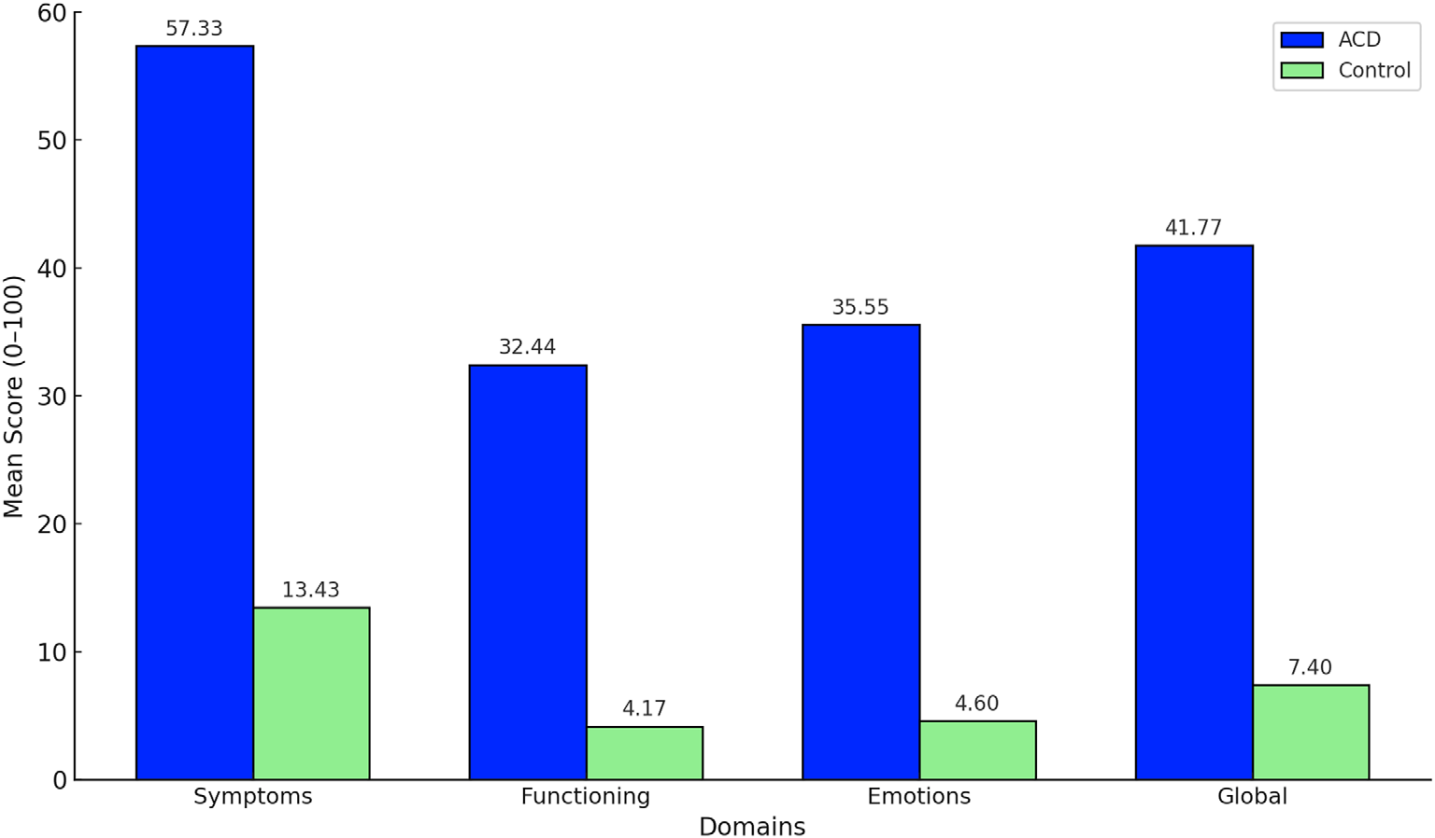
Comparison of mean scores between the ACD and control groups in the Skindex‐29 subscales: symptoms, emotions, functioning and global index.

The Skindex‐29 scores were also significantly higher in the ACD group across all domains: Symptoms (mean = 57.33), Functioning (mean = 32.44), Emotions (mean = 35.55) and Global Index (mean = 41.77), compared with controls (means = 13.43, 4.17, 4.60 and 7.40, respectively), with all differences reaching statistical significance (*p* < 0.001). Figure 4 illustrates the distribution of scores across Skindex‐29 domains, further highlighting the QoL burden in ACD.

Item‐level analysis (Table S2; Figure S2) revealed that all items were significantly more impaired in ACD patients (*p* < 0.0001), with the highest scores observed for itching, burning, water sensitivity and feelings of humiliation or frustration.

### 3.3 Sociodemographic Determinants of Health‐Related Quality of Life in Patients With Allergic Contact Dermatitis

#### 3.3.1 Sex

Analysis of the Skindex‐29 revealed that female patients reported a significantly greater symptom burden than males on the Symptoms subscale (mean = 59.77, SD = 24.80 vs. 52.25, SD = 21.86; *p* < 0.05). No sex‐related differences were observed in the Emotions, Functioning, or Global Index subscales. No significant differences were found in DLQI or EQ‐5D index scores between men and women.

#### 3.3.2 Age

DLQI scores showed a positive relation with age in linear regression analysis, indicating that older patients reported greater impairment. Conversely, EQ‐5D index scores exhibited a significant negative correlation with age, reflecting poorer perceived health status in older individuals. On the Skindex‐29, age was significantly associated with increased functional limitation (*p* = 0.039), but no associations were found with the other subscales.

#### 3.3.3 Marital Status

No significant differences were observed in DLQI, EQ‐5D, or any of the Skindex‐29 subscales based on marital status.

#### 3.3.4 Educational Level

Educational level was not significantly associated with DLQI or EQ‐5D scores. However, Skindex‐29 results showed a significant inverse relationship between educational attainment and QoL. Lower levels of education were associated with higher (worse) scores on the Emotions (*p* = 0.029), Functioning (*p* = 0.007), and Global Index (*p* = 0.025) subscales.

#### 3.3.5 Disease Severity

Although ANOVA did not detect differences in DLQI scores across severity categories, linear regression analysis revealed that disease severity (measured by mIGA) was a significant predictor of higher DLQI scores. EQ‐5D scores were not significantly associated with disease severity. In contrast, Skindex‐29 subscales revealed that greater disease severity was significantly associated with higher scores on the Emotions (*p* = 0.022), Functioning (*p* = 0.017) and Global Index (*p* = 0.013) subscales. The association with the Symptoms subscale approached statistical significance (*p* = 0.053).

#### 3.3.6 Disease Duration and Emergency Department Visits

Longer disease duration correlated with higher symptom burden on the Skindex‐29 Symptoms subscale (*p* = 0.045), without significant effects on the other dimensions. However, disease duration was not significantly associated with DLQI or EQ‐5D scores. The number of emergency department visits in the past year showed no significant associations with DLQI, EQ‐5D, or any Skindex‐29 subscales.

#### 3.3.7 Most Frequently Reported Symptoms

According to Skindex‐29 responses, pruritus was reported by 45.0% of patients with ACD, with 42.7% rating the intensity as moderate and 21.8% as severe. Other frequently reported complaints included skin irritation (34.2%) and increased sensitivity (38.2%). Functional impact included moderate work impairment in 33.3% and disruption of social activities in 31.6% of patients.

## 4 Discussion

This study demonstrates that patients with ACD experience significantly reduced QoL compared to healthy controls. By simultaneously applying three validated instruments, DLQI, Skindex‐29 and EQ‐5D‐5L, our findings provide a comprehensive picture of the multidimensional burden of ACD, including symptoms, emotional distress, functional impairment and overall health status. The Skindex‐29 functioning domain emerged as the strongest contributor to global impairment, underscoring the significant disruption that ACD imposes on daily activities.

Our item‐level analysis of DLQI scores further illustrates the wide‐ranging effect of ACD on patients' lives, emphasising that not only physical symptoms but also emotional and functional aspects are markedly impaired. This granular approach provides complementary insight into which specific daily‐life activities and emotional factors are most affected, aligning with previous reports highlighting the psychological and social burden of ACD.

A similar item‐level exploration of the Skindex‐29 provided an even more detailed understanding of the symptom patterns most strongly associated with impaired QoL. The highest item scores corresponded to itching, burning, or stinging sensations, water sensitivity and feelings of humiliation and frustration. These findings confirm that sensory symptoms and emotional distress are key drivers of overall burden in ACD and contribute to the loss of social and occupational functioning. Such detailed analyses support the need for multidimensional therapeutic approaches that address both cutaneous symptoms and psychosocial sequelae, integrating pruritus control and psychological support into patient management.

Consistent with previous literature [22], our results showed that female patients reported higher symptom burden, particularly on the Skindex‐29 Symptoms subscale, although no sex‐related differences were observed in overall QoL scores. Age was also significantly associated with reduced QoL, with older patients reporting greater impairment across the DLQI, EQ‐5D‐5L and the Skindex‐29 Functioning subscale. This trend may partly reflect the general decline in health‐related QoL with ageing, but it also suggests a potentially synergistic effect between age and ACD burden. Conversely, marital status and educational level showed no consistent associations with QoL, aligning with prior research indicating that these sociodemographic variables exert limited influence in the context of ACD [23].

Disease severity, as assessed by mIGA, emerged as a significant predictor of QoL impairment across multiple domains, reinforcing its clinical value as a core measure in ACD management [24, 25]. However, severity alone does not fully capture the burden experienced by patients. ACD is a multifactorial condition, where additional elements such as lesion visibility, occupational exposure and chronic disease course also play a critical role in diminishing QoL [26, 27]. For instance, Jampuram et al. reported moderate QoL impairment in patients with cosmetic dermatitis, underscoring the specific impact of facial involvement and sensitizers such as para‐phenylenediamine [28]. In occupational settings, the perceived burden may paradoxically be lower despite high allergen exposure; among 340 beekeepers sensitised to propolis, the mean DLQI score remained relatively modest [29]. Thus, although the mIGA is a valuable and practical tool for evaluating clinical severity, it should be incorporated into a broader multidimensional assessment framework to reflect more accurately the overall impact of ACD on patients' lives.

Interestingly, emergency department visits were not associated with QoL scores, despite being frequent. This may reflect suboptimal ACD management in emergency care settings, where lack of patch testing, misdiagnosis and limited follow‐up contribute to persistent symptoms and emotional distress [30]. Our findings reinforce the value of early dermatologic intervention and allergen identification via patch testing, which can substantially improve disease control and reduce reliance on emergency services [31]. As seen in psoriasis, patient satisfaction and adherence are closely tied to improved QoL outcomes [32], which may also apply to ACD. This highlights the need for integrating validated QoL measures into routine ACD care.

Patient‐reported outcomes are increasingly prioritised in dermatology, yet remain underutilised in contact dermatitis. The HECOS project on hand eczema proposed core domains for PROs, including pruritus, pain and occupational impact [33]. While tools such as the Skindex‐29 and patient global assessments (PtGA) are recommended [34, 35], no universally accepted patient‐reported outcomes exist for ACD [36]. A recent consensus recommended the systematic assessment of mood, sleep and self‐esteem in patients with chronic dermatoses [37]. However, studies indicate that instruments such as the DLQI and Skindex‐29 remain underutilised in routine clinical practice by Spanish dermatologists, even in conditions like psoriasis [38].

Several QoL tools have been developed specifically for ACD [39], including the ACD‐Index [40, 41], CDQL [42], FQL Index [43] and QOLHEQ [44]. However, none have been validated in Spanish, limiting their utility. Cultural adaptation efforts, such as the RosaQoL in rosacea [45], demonstrate the value of such tools in capturing disease burden and monitoring outcomes [46]. Our results support the need to develop and validate a disease‐specific, Spanish‐language QoL instrument for ACD.

In this context, the simultaneous use of DLQI, Skindex‐29 and EQ‐5D‐5L in our study provided complementary insights into dermatology‐specific, emotional and global health domains, offering a more nuanced understanding of the patient experience. This multidimensional approach may enhance clinical decision‐making and support its adoption in routine care. Furthermore, given the lack of validated Spanish‐language instruments specific to ACD, our findings could contribute to the cross‐cultural adaptation and future validation of tools such as the ACD‐Index for use in Spanish‐speaking populations.

This study has several limitations. Its cross‐sectional design prevents causal inference. Additionally, recruitment from a single tertiary centre and the use of a convenience sampling approach for control selection may reduce the generalizability of the findings. However, this strategy ensured sociodemographic comparability between groups and is consistent with previous QoL research in dermatology. However, the inclusion of a healthy control group allowed for the identification of significant differences; comparing ACD patients to individuals with other chronic dermatologic conditions could have provided more context regarding disease‐specific burden. Lastly, while all instruments used were validated in Spanish, the absence of a disease‐specific QoL questionnaire tailored to ACD limits the precision with which the psychosocial impact of this condition can be assessed.

ACD is a chronic inflammatory condition with a substantial psychosocial burden. Routine assessment of QoL can facilitate early identification of emotional distress and functional impairment, supporting a more patient‐centred and holistic approach to care. Although our study did not evaluate psychological comorbidities or sleep disturbances directly [47], these factors are frequently reported in ACD and warrant further investigation. Similarly, the economic impact of ACD, related to productivity loss, treatment costs and healthcare utilisation, deserves future research attention. Integrating psychological support within multidisciplinary contact dermatitis units may further enhance patient outcomes.

In conclusion, ACD significantly impairs QoL, especially among patients with higher disease severity, highlighting the need for timely diagnosis and targeted therapeutic strategies. To enhance patient‐centred care, the use of multidimensional assessments and culturally adapted tools tailored to ACD is essential. Moreover, our findings support the integration of QoL measures and patient‐reported outcomes into clinical guidelines to ensure comprehensive management of ACD.

## Author Contributions

**Álvaro Prados‐Carmona:** writing – original draft, writing – review and editing. **Ricardo Ruiz‐Villaverde:** writing – original draft, writing – review and editing, supervision. **Francisco José Navarro‐Triviño:** conceptualization, investigation, writing – original draft, methodology, validation, visualization, writing – review and editing, formal analysis. **María Isabel Peralta‐Ramírez:** writing – original draft, writing – review and editing, supervision.

## Funding

Funding for open access charge: Universidad de Granada/CBUA.

## Conflicts of Interest

The authors declare no conflicts of interest.

## Supporting information


**Figure S1:** Item‐level comparison of Dermatology Life Quality Index (DLQI) scores between patients with allergic contact dermatitis (ACD) and healthy controls. Bars represent mean scores ± standard deviation for each of the 10 DLQI items. Items correspond to the domains of symptoms, feelings, daily activities, leisure, work/school, personal relationships and treatment. *p* < 0.001 for all items (Mann–Whitney U test).

**Figure S2:** Item‐level comparison of Skindex‐29 scores between patients with allergic contact dermatitis (ACD) and healthy controls. Bars represent mean scores ± standard deviation for each of the 29 Skindex‐29 items. The greatest differences between groups were observed in the symptoms (itching, burning, pain) and emotions (embarrassment, frustration, humiliation) domains, followed by functioning (interference with daily and social activities). *p* < 0.001 for all items (Mann–Whitney U test).

**Table S1:** Dermatology Life Quality Index (DLQI) item‐level analysis in patients with allergic contact dermatitis (ACD) and healthy controls. Values are expressed as mean ± standard deviation. Comparisons between groups were made using the Mann–Whitney U test. *p* < 0.05 was considered statistically significant.
**Table S2:** Skindex‐29 item‐level analysis in patients with allergic contact dermatitis (ACD) and healthy controls. Values are expressed as mean ± standard deviation. All comparisons were statistically significant (*p* < 0.001, Mann–Whitney U test). Items are ordered according to their official wording in the validated English version of Skindex‐29.

## Data Availability

The data that support the findings of this study are available from the corresponding author upon reasonable request.
