# Supplementary figures and images for: Impact of Allergic Contact Dermatitis on Health‐Related Quality of Life: A Cross‐Sectional Case–Control Study in a Spanish Population
Source: Contact Dermatitis. 2026 Feb 22;94(6):592–602. doi: 10.1111/cod.70116 (PMC13139706; doi:10.1111/cod.70116)

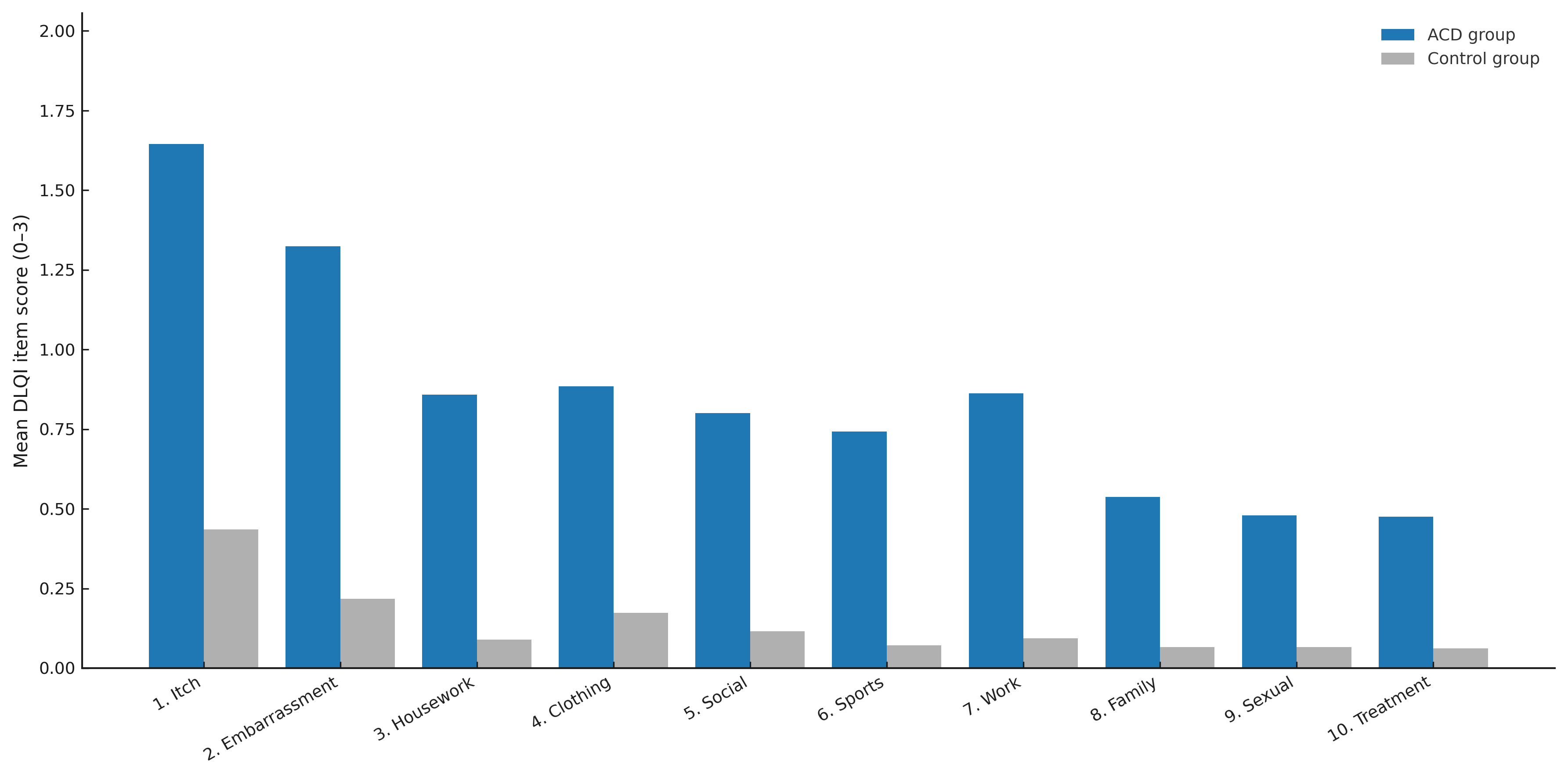

Supplement: Supplementary file 1 — Figure S1: Item‐level comparison of Dermatology Life Quality Index (DLQI) scores between patients with allergic contact dermatitis (ACD) and healthy controls. Bars represent mean scores ± standard deviation for each of the 10 DLQI items. Items correspond to the domains of symptoms, feelings, daily activities, leisure, work/school, personal relationships and treatment. p < 0.001 for all items (Mann–Whitney U test). [file COD-94-592-s001.png]

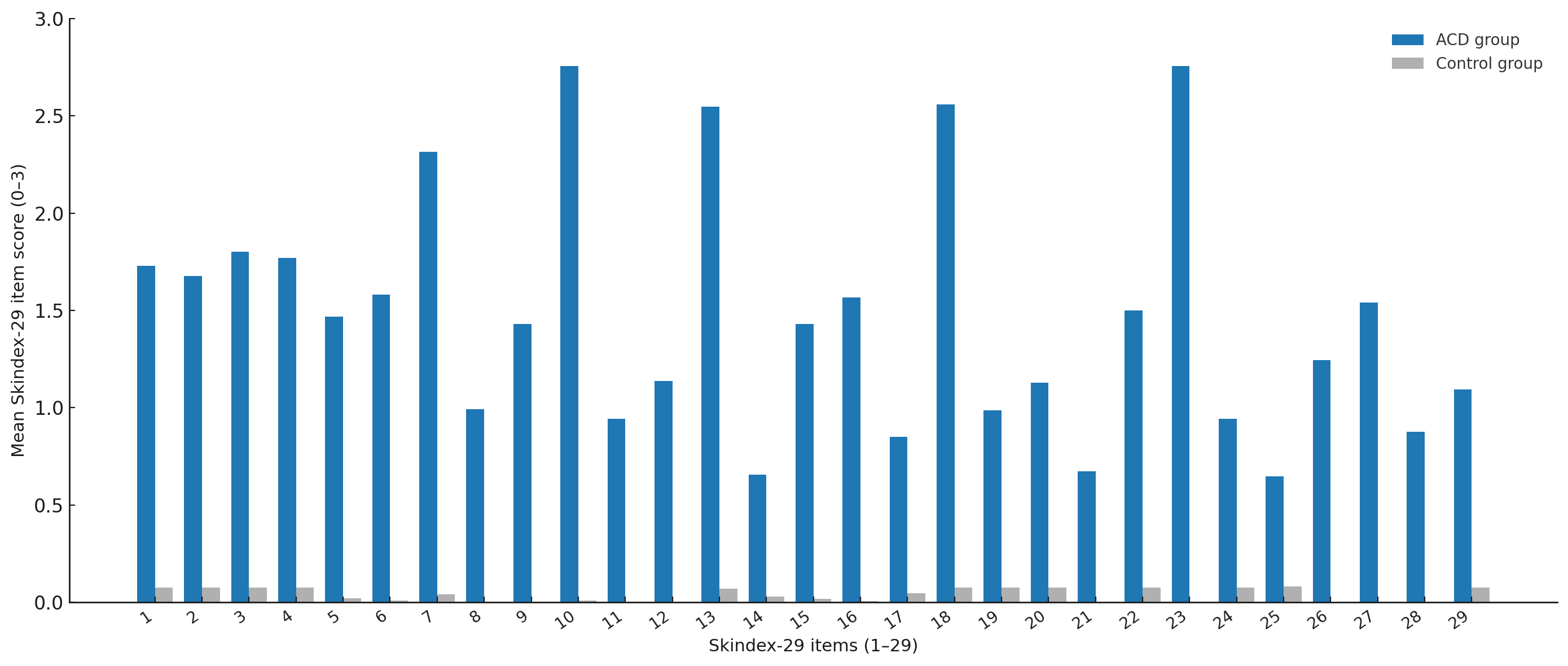

Supplement: Supplementary file 2 — Figure S2: Item‐level comparison of Skindex‐29 scores between patients with allergic contact dermatitis (ACD) and healthy controls. Bars represent mean scores ± standard deviation for each of the 29 Skindex‐29 items. The greatest differences between groups were observed in the symptoms (itching, burning, pain) and emotions (embarrassment, frustration, humiliation) domains, followed by functioning (interference with daily and social activities). p < 0.001 for all items (Mann–Whitney U test). [file COD-94-592-s002.png]
