# Supplementary material for: Impact of Allergic Contact Dermatitis on Health‐Related Quality of Life: A Cross‐Sectional Case–Control Study in a Spanish Population
Source: Contact Dermatitis. 2026 Feb 22;94(6):592–602. doi: 10.1111/cod.70116 (PMC13139706; doi:10.1111/cod.70116)
Supplement: Supplementary file 3 — Table S1: Dermatology Life Quality Index (DLQI) item‐level analysis in patients with allergic contact dermatitis (ACD) and healthy controls. Values are expressed as mean ± standard deviation. Comparisons between groups were made using the Mann–Whitney U test. p < 0.05 was considered statistically significant. Table S2: Skindex‐29 item‐level analysis in patients with allergic contact dermatitis (ACD) and healthy controls. Values are expressed as mean ± standard deviation. All comparisons were statistically significant (p < 0.001, Mann–Whitney U test). Items are ordered according to their official wording in the validated English version of Skindex‐29. [file COD-94-592-s003.docx]

Supplementary Table S1. Dermatology Life Quality Index (DLQI) item-level analysis in patients with allergic contact dermatitis (ACD) and healthy controls. Values are expressed as mean ± SD. Comparisons between groups were made using the Mann–Whitney U test. *p* < 0.05 was considered significant.

| **Item no.** | **DLQI item (short English wording)** | **Mean ± SD (ACD)** | **Mean ± SD (Control)** | **Mean difference** | **p-value** |
| --- | --- | --- | --- | --- | --- |
| 1 | Itchy, sore, painful, or stinging skin | 1.69 ± 1.10 | 0.48 ± 0.65 | 1.21 | <0.0001 * |
| 2 | Feeling embarrassed or self-conscious | 1.55 ± 1.01 | 0.44 ± 0.61 | 1.11 | <0.0001 * |
| 3 | Interference with shopping, housework, or gardening | 1.02 ± 0.88 | 0.25 ± 0.46 | 0.77 | <0.0001 * |
| 4 | Choice of clothing affected | 0.91 ± 0.89 | 0.20 ± 0.45 | 0.71 | <0.0001 * |
| 5 | Social or leisure activities affected | 0.86 ± 0.83 | 0.20 ± 0.45 | 0.66 | <0.0001 * |
| 6 | Difficulty with sports or hobbies | 0.78 ± 0.82 | 0.16 ± 0.39 | 0.62 | <0.0001 * |
| 7 | Problems at work or school | 0.77 ± 0.82 | 0.18 ± 0.42 | 0.59 | <0.0001 * |
| 8 | Difficulties with partner, family, or close friends | 0.65 ± 0.79 | 0.10 ± 0.31 | 0.55 | <0.0001 * |
| 9 | Sexual difficulties | 0.45 ± 0.68 | 0.07 ± 0.27 | 0.38 | <0.0001 * |
| 10 | Problems caused by treatment (time, mess, etc.) | 0.70 ± 0.78 | 0.16 ± 0.38 | 0.54 | <0.0001 * |
|  | Total DLQI score | 8.72 ± 6.80 | 1.39 ± 3.57 | 7.33 | <0.0001 * |

* Significant difference between ACD and control groups (*p* < 0.05, Mann–Whitney U).

Supplementary Table S2. **S**kindex-29 item-level analysis in patients with allergic contact dermatitis (ACD) and healthy controls. Values are expressed as mean ± SD. All comparisons were significant (*p* < 0.0001, Mann–Whitney U test). Items are ordered according to their official wording in the validated English version of Skindex-29.

| **Item no.** | **Skindex-29 item (official English wording)** | **Mean ± SD (ACD)** | **Mean ± SD (Control)** | **Mean difference** | **p-value** |
| --- | --- | --- | --- | --- | --- |
| 1 | My skin hurts | 1.73 ± 1.15 | 0.08 ± 0.27 | 1.65 | <0.0001 * |
| 2 | My skin condition affects my sleep | 1.68 ± 1.17 | 0.08 ± 0.27 | 1.60 | <0.0001 * |
| 3 | I worry that my skin condition may be serious | 1.80 ± 1.20 | 0.08 ± 0.27 | 1.72 | <0.0001 * |
| 4 | My skin condition interferes with my work or hobbies | 1.77 ± 1.25 | 0.08 ± 0.27 | 1.69 | <0.0001 * |
| 5 | My skin condition affects my social life | 1.47 ± 1.15 | 0.02 ± 0.14 | 1.45 | <0.0001 * |
| 6 | My skin condition depresses me | 1.58 ± 1.18 | 0.01 ± 0.10 | 1.57 | <0.0001 * |
| 7 | My skin burns or stings | 2.31 ± 1.14 | 0.04 ± 0.20 | 2.27 | <0.0001 * |
| 8 | I tend to stay at home because of my skin condition | 0.99 ± 1.11 | 0.00 ± 0.00 | 0.99 | <0.0001 * |
| 9 | I worry that my skin condition will leave scars | 1.43 ± 1.30 | 0.00 ± 0.00 | 1.43 | <0.0001 * |
| 10 | My skin itches | 2.76 ± 0.95 | 0.01 ± 0.10 | 2.75 | <0.0001 * |
| 11 | My skin condition affects my interactions with people | 0.94 ± 1.17 | 0.00 ± 0.00 | 0.94 | <0.0001 * |
| 12 | My skin condition affects my relationship with loved ones | 1.14 ± 1.27 | 0.00 ± 0.00 | 1.14 | <0.0001 * |
| 13 | I worry that my skin condition will get worse | 2.55 ± 1.05 | 0.07 ± 0.25 | 2.48 | <0.0001 * |
| 14 | I tend to do things alone because of my skin condition | 0.65 ± 0.99 | 0.03 ± 0.17 | 0.63 | <0.0001 * |
| 15 | I am angry about my skin condition | 1.43 ± 1.25 | 0.02 ± 0.13 | 1.41 | <0.0001 * |
| 16 | I am frustrated by my skin condition | 1.57 ± 1.35 | 0.00 ± 0.07 | 1.56 | <0.0001 * |
| 17 | I have difficulty showing affection because of my skin condition | 0.85 ± 1.13 | 0.05 ± 0.21 | 0.80 | <0.0001 * |
| 18 | Water worsens my skin condition | 2.56 ± 1.09 | 0.08 ± 0.27 | 2.48 | <0.0001 * |
| 19 | My skin is irritated | 0.99 ± 1.14 | 0.08 ± 0.27 | 0.91 | <0.0001 * |
| 20 | My skin condition causes embarrassing situations | 1.13 ± 1.11 | 0.08 ± 0.27 | 1.05 | <0.0001 * |
| 21 | I am ashamed of my skin condition | 0.67 ± 1.07 | 0.00 ± 0.00 | 0.67 | <0.0001 * |
| 22 | My skin is sensitive | 1.50 ± 1.33 | 0.08 ± 0.27 | 1.42 | <0.0001 * |
| 23 | I find my skin condition humiliating | 2.76 ± 0.95 | 0.00 ± 0.00 | 2.76 | <0.0001 * |
| 24 | My skin condition affects my desire to be with people | 0.94 ± 1.16 | 0.08 ± 0.27 | 0.87 | <0.0001 * |
| 25 | My skin condition affects my sex life | 0.65 ± 1.05 | 0.08 ± 0.27 | 0.57 | <0.0001 * |
| 26 | My skin condition bleeds | 1.24 ± 1.11 | 0.00 ± 0.00 | 1.24 | <0.0001 * |
| 27 | My skin condition is a problem for people I love | 1.54 ± 1.20 | 0.00 ± 0.00 | 1.54 | <0.0001 * |
| 28 | I am bothered by the appearance of my skin condition | 0.88 ± 1.15 | 0.00 ± 0.00 | 0.88 | <0.0001 * |
| 29 | My skin condition makes me tired | 1.09 ± 1.19 | 0.08 ± 0.27 | 1.02 | <0.0001 * |
